# Ovarian reserve parameters and IVF outcomes in 510 women with poor ovarian response (POR) treated with intraovarian injection of autologous platelet rich plasma (PRP)

**DOI:** 10.18632/aging.203972

**Published:** 2022-03-22

**Authors:** Yigit Cakiroglu, Aysen Yuceturk, Ozge Karaosmanoglu, Sule Yildirim Kopuk, Zeynep Ece Utkan Korun, Nola Herlihy, Richard T. Scott, Bulent Tiras, Emre Seli

**Affiliations:** 1 Acibadem Maslak Hospital Assisted Reproductive Technologies Unit, Sariyer, Istanbul, Turkey; 2 Acibadem Mehmet Ali Aydinlar University, Department of Obstetrics and Gynecology, Sariyer, Istanbul, Turkey; 3 IVI RMA New Jersey, Basking Ridge, NJ 07920, USA; 4 Department of Obstetrics and Gynecology, Thomas Jefferson University, Philadelphia, PA 19107, USA; 5 Department of Obstetrics, Gynecology and Reproductive Sciences, Yale School of Medicine, New Haven, CT 06510, USA

**Keywords:** poor ovarian response, platelet rich plasma, *in vitro* fertilization

## Abstract

The aim of the current study was to characterize ovarian reserve parameters and IVF outcomes in women with a history of poor ovarian response (POR) treated with intraovarian injection of autologous platelet rich plasma (PRP). Reproductive age women (N=510; age range 30-45yo) diagnosed with POR based on Poseidon criteria were included in the study. PRP treatment resulted in higher AFC, higher serum AMH, lower serum FSH, and a higher number of mature oocytes and cleavage and blastocyst stage embryos. After PRP injection, 22 women (4.3%) conceived spontaneously, 14 (2.7%) were lost to follow up, and 474 (92.9%) attempted IVF. Among women who attempted IVF, 312 (65.8%) generated embryos and underwent embryo transfer, 83 (17.5%) achieved a pregnancy, and 54 (11.4%) achieved sustained implantation/live birth (SI/LB). In total, of the 510 women with POR and mean age of 40.3, PRP resulted in improvement of ovarian reserve parameters, a pregnancy rate of 20.5% and SI/LB rate of 12.9%. Our findings suggest that PRP treatment may be considered in women with POR. For wider clinical application, its clinical efficacy will need to be demonstrated in prospective randomized clinical trials.

## INTRODUCTION

Ovarian aging is a physiological process associated with a decline in oocyte quantity and quality [1]. It has critical implications for fertility and is an increasingly more prevalent reason for women to seek fertility treatment [2]. It is not only the decreasing number, but also the worsening quality of oocytes that contributes to impaired fertility outcomes in aging women, including decreased fertilization and blastocyst formation rates and higher aneuploidy rates [3].

A subset of the infertile population demonstrate accelerated ovarian aging. These women are labeled “poor ovarian response” (POR) or “poor responders” due to a combination of low parameters of ovarian reserve and previous low oocyte yield after ovarian stimulation. In order to standardize the definition of POR, the European Society for Human Reproduction and Infertility (ESHRE) proposed that two of the following three criteria would lead to a POR diagnosis: (i) advanced maternal age (≥40yo) or any other risk factor for POR; (ii) a previous cycle with ≤3 oocytes retrieved using conventional stimulation; (iii) an abnormal ovarian reserve test (i.e. AFC, 5–7 follicles or AMH, 0.5–1.1 ng/ml) [4]. Subsequently, the terminology used to characterize these patients evolved from POR to “low prognosis” with the introduction of the POSEIDON criteria, a more nuanced classification system that reflects the heterogeneity in the population [5].

Women with a POR diagnosis account for 15% of all assisted reproductive technology (ART) cycles performed in the United States. Treating these patients poses a significant challenge for reproductive endocrinologists, as their cycles are more likely to be cancelled or result in lower number of embryos available for transfer and lower pregnancy rates [6–9]. Consequently, a number of experimental approaches have been tested in this population in order to promote follicle activation, increase the number of eggs retrieved and improve IVF outcomes. Ovarian fragmentation for *in vitro* activation (IVA) in combination with Akt-stimulating drugs was first suggested by Kawamura et al. in women with primary ovarian insufficiency, and multiple subsequent studies have demonstrated encouraging results [10–12]. Ovarian fragmentation increases actin polymerization leading to an interruption in the intracellular Hippo signaling, which, in turn, increases cell proliferation and promotes activation of primordial follicles [13]. Another experimental procedure, autologous stem cell ovarian transplantation (ASCOT) has also been tested in poor responders and resulted in improved ovarian function and increased number of antral follicles and oocytes [14]. These novel interventions, while promising, are highly invasive and unproven in randomized clinical trials.

Another less invasive approach towards improving ovarian response for poor responders is intra-ovarian injection of platelet rich plasma (PRP). PRP is derived via centrifugation of whole blood, and is rich in growth factors and cytokines. Several of these factors promote healing and tissue regeneration by inducing chemotaxis, cell migration and differentiation. Moreover, they contribute to angiogenesis and inflammatory changes, which play key roles in tissue repair and regeneration [15, 16]. PRP also promotes follicle development *in vitro* and small case series have demonstrated that it may be an effective treatment for women with POR [17–19]. The aim of the current study was to characterize ovarian reserve parameters and IVF outcomes in a large cohort of 510 women with POR treated with intraovarian injection of autologous PRP.

## RESULTS

**Figure 1:**
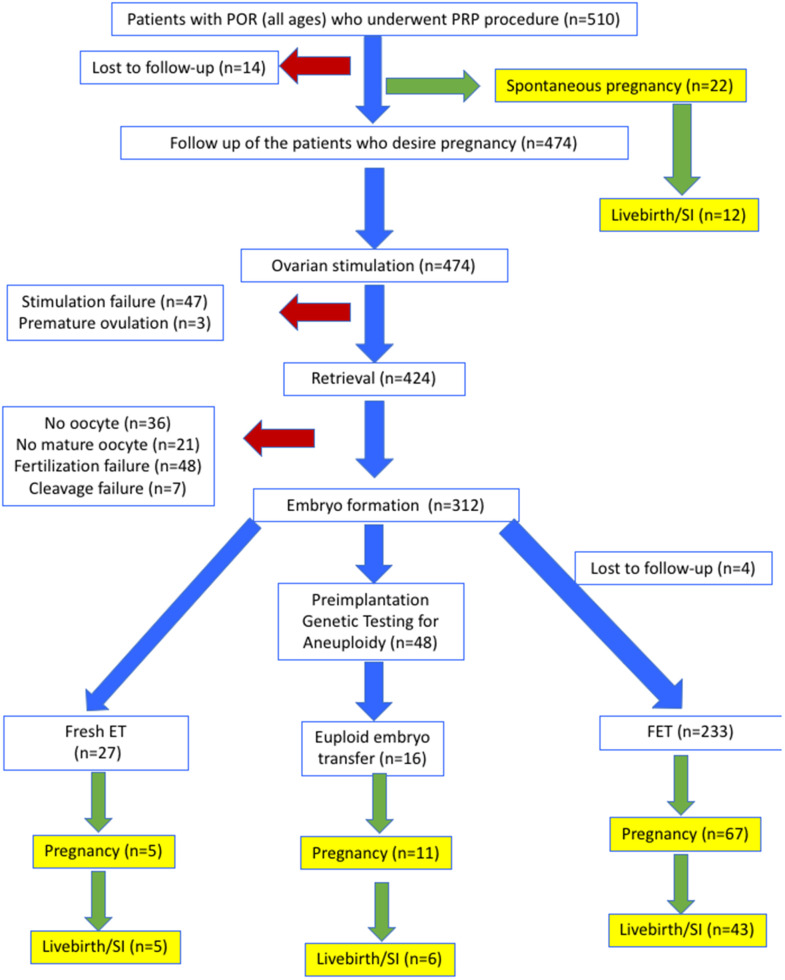
Flowsheet of outcomes for all patients.

A total of 510 women (mean age ± SD: 40.3 ± 4.0) with the diagnosis of POR were included in the study. Flowcharts of outcomes are shown in Figure 1.

### Spontaneous pregnancies in response to PRP

Spontaneous pregnancy occurred in 22 women (4.3%, mean age ± SD: 39.1 ± 4.4), one to seven cycles (mean ± SD: 2.4 ± 1.6) after the PRP procedure (Figure 1). Characteristics of these women are presented in Supplementary Table 1. At the time of this report, 10 of the spontaneously conceived pregnancies were lost as spontaneous miscarriages, while 4 were ongoing between 16th to 23rd weeks of gestation and 8 were delivered between 32nd to 39th weeks of gestation. Therefore, 12/22 (54.5%) of spontaneous pregnancies that developed after PRP treatment resulted in sustained implantation or livebirth (2.3% of women who received PRP).

### Ovarian reserve assessment

When ovarian reserve parameters were analyzed, we observed a statistically significant increase in AFC following PRP treatment (4.2 ± 2.4 vs 2.6 ± 1.3; p<0.001). Serum AMH also increased after PRP treatment (0.53 ± 0.39 vs 0.35 ± 0.32; p<0.001), with a decrease in serum FSH (16.4 ± 14.0 vs 20.6 ± 18.3; p<0.001) (Table 1). Time to antral follicle detection after PRP was calculated as 2.5 ± 0.9 cycles (min-max: 2-7). When the number of follicle waves was analyzed in order to estimate the duration of positive effects of PRP on ovarian reserve, we calculated as 1.6 ± 0.9 cycles/patient (min-max: 1-5 waves).

**Table 1:** Antral follicle count, FSH, and AMH parameters of women with POR who underwent intraovarian autologous PRP injection (mean ± SD).

	**Before PRP**	**After PRP**	***p***
Number of IVF cycles	2.8 ± 2.9	1.6 ± 0.9	<0.001
Antral follicle count (n)	2.6 ± 1.3	4.2 ± 2.4	<0.001
FSH (IU/mL)	20.6 ± 18.3	16.4 ± 14.0	<0.001
AMH (ng/mL)	0.35 ± 0.32	0.53 ± 0.39	<0.001
Number of retrieved oocytes (in women who had oocytes retrieved; n=388)	2.2 ± 1.9	3.4 ± 2.7	<0.001
Number of mature oocytes (in women who had mature oocytes retrieved; n=367)	1.7 ± 1.4	2.7 ± 2.0	<0.001
Number of 2 pronuclei embryos (in women with fertilization; n=319)	1.3 ± 1.2	2.1 ± 1.7	<0.001
Fertilization rate (%) (in women with fertilization; n=319)	57.6	66.9	0.008
Number of cleavage stage embryos (in women with fertilization; n=312)	1.3 ± 1.1	2.3 ± 1.5	<0.001
Number of blastocysts (in women with fertilization; n=18)	0.6 ± 0.9	2.3 ± 1.6	<0.001

### IVF outcomes

After excluding 22 patients with spontaneous conception and 14 patients who were lost to follow up, 474 women were candidates for IVF and underwent COH. IVF cycle was initiated after a mean of 2.6 ± 0.9 cycles (min-max: 2-7). Clinical and IVF outcome parameters of women with POR who underwent intraovarian autologous PRP injection are demonstrated in Supplementary Table 2.

Oocyte retrieval was performed in 424 (89.5% of stimulated) women, while 50 (10.5%) could not undergo oocyte retrieval due to either stimulation failure (n=47) or premature ovulation (n=3) (Figure 1). Among the women who underwent oocyte retrieval, 367 (86.6%) achieved at least one mature oocyte. Mean number of oocytes per retrieval before and after PRP were 2.2 ± 1.9 and 3.4 ± 2.7 (p<0.001), respectively. In 312 women (65.8% of stimulated), at least one cleavage stage embryo was obtained. The mean number of 2PN, and cleavage stage embryos obtained before and after PRP were, 1.3 ± 1.2 and 2.1 ± 1.7 (p<0.001) respectively, and 1.3 ± 1.1 and 2.3 ± 1.5 (p<0.001), respectively. The mean number of blastocysts obtained before and after PRP were, 0.6 ± 0.9 and 2.3 ± 1.6 (p<0.001). The mean duration of time to embryo generation in case of FET was 3.2 ± 1.5 cycles (min-max: 2-8).

Of the 312 women who developed embryos, 260 underwent fresh or frozen ET without PGT-A, while 48 opted for PGT-A. Among the 260 women who underwent IVF without PGT-A, 5/27 (18.5%) of fresh ETs, and 67/233 (28.8%) FETs resulted in pregnancy. All five pregnancies from fresh ETs (n=5) were achieved after a mean of 2.8 ± 1.3 cycles (min-max: 2-5 cycles) after PRP and resulted in live births. Of the 67 FET pregnancies, 26 (38.8%) miscarried during the first trimester, 18 (26.9%) were ongoing between 17 and 36 weeks of gestation, and 23 (34.3%) resulted in live birth. Among the 48 women who underwent PGT-A, the mean age was 40.2 ± 3.6 (min-max: 31-46) and 16 had euploid embryos. The overall euploidy rate before and after PRP were 11.7% (4/34 embryos) and 16.8% (20/119 embryos) respectively. FET, resulted in 11 pregnancies (68.8%) and six live births (37.5%). In total, of the women who developed embryos after PRP treatment, 83/312 (26.6%) achieved pregnancy, and 54/312 (17.3%) achieved sustained implantation or livebirth. In the overall population, the cumulative pregnancy rate was 21.2% (105/496), and the cumulative SI/LB was 13.3% (66/496) after exclusion of lost-to follow-up patients.

### IVF outcomes in age subgroups

As expected, IVF outcome parameters in women treated with PRP were affected by patient age. Subgroup assessment was performed dividing patients into three groups (<38yo, 38 to 42yo; 42 to 45yo). Comparison of IVF and pregnancy outcomes according to age subgroups are demonstrated in Table 2.

**Table 2:** Comparison of IVF and pregnancy outcomes according to age subgroups. Values are expressed as number (n) (percentage; %).

	**<38yo (n=132)**	**38-42yo (n=192)**	**42-45yo (n=186)**
Spontaneous pregnancy n (%)	9 (6.8)	8 (4.2)	5 (2.7)
Lost to follow up n (%)	3 (2.3)	8 (4.2)	3 (1.6)
Developed embryos n (%)	96 (72.7)	113 (58.8)	103 (55.3)
Achieved pregnancy n (%)	58 (45)	28 (15.2)	6 (3.2)
Sustained pregnancy/livebirth n (%)	44 (34.1)	16 (8.7)	2 (1)

In addition, a receiver operating characteristic (ROC) curve analysis was performed and 40 years old (sensitivity: 48.35, specificity: 70.93; AUC:0.612) was calculated as a cut-off for the patients who would not benefit from PRP due to absence of ovarian response. Similarly, patients >38 yo (sensitivity: 61.54, specificity: 73.77; AUC:0.705) had higher risk of miscarriage rates.

### Baseline ovarian reserve parameters, ovarian volume, PRP injection techniques and response to treatment

We assessed serum FSH and AMH levels, and AFC at baseline (prior to PRP injection) in association with the likelihood of producing at least one fertilized embryo. We found that levels of FSH, AMH and AFC were statistically significantly different in women who generated at least one fertilized embryo (Table 3). Cut off levels for FSH, AMH, and AFC were 21.2 mIU/ml (Sens:75.45, spec:42.59; AUC:0.619), 0.23 ng/ml (Sens:65.57, spec:64.20; AUC:0.670) and 1 (Sens:80.84, spec:38.89; AUC:0.627), respectively.

**Table 3:** Predictive factors for PRP related procedures (ovarian volumes, number of punctures, PRP volume) compared according to obtaining one fertilized embryo (mean ± SD).

	**At least one fertilized embryo**	**No fertilized embryo**	***p***
FSH (IU/mL)	17.8 ± 13.5	26.2 ± 24.2	<0.001*
AMH (ng/mL)	0.40 ± 0.34	0.24 ± 0.22	<0.001*
AFC	2.8 ± 1.3	2.2 ± 1.2	<0.001*
Right ovary volume (cm^3^)	5.41 ± 5.18	3.87 ± 2.67	<0.001*
Left ovary volume (cm^3^)	5.32 ± 4.62	4.15 ± 3.75	0.06*
Mean volume (cm^3^)	5.36 ± 3.65	3.98 ± 2.54	<0.001*
Mean number of punctures (n)	3.3 ± 0.6	3.3 ± 0.6	0.81
Total PRP amount injected into both ovaries (ml)	6.9 ± 2.5	6.8 ± 1.1	0.56
Mean PRP amount injected into each ovary (ml)	3.5 ± 1.2	3.4 ± 0.8	0.86

We also investigated the relationship between ovarian volume, PRP injection volume, and number of ovarian punctures and the likelihood of developing at least one embryo. Our data revealed a statistically significant relationship between mean ovarian volume and probability of having at least one fertilized embryo (cut-off value: 4.30 cm^3^ (Sens:52.9, spec:69.4; AUC=0.624)). Injected PRP volume and the number of ovarian punctures did not differ between patients who developed at least one fertilized embryo and those who did not.

## DISCUSSION

In this study, we investigated whether intraovarian injection of PRP improves ovarian reserve parameters and IVF outcomes in women with POR. Intraovarian PRP injection was performed in a total of 510 women with a mean age of 40.3. This intervention resulted in improvement of ovarian reserve parameters, a pregnancy rate of 20.5% and a sustained implantation/livebirth rate of 12.9%.

PRP has been investigated and implemented into routine clinical practice in many fields of medicine as a rejuvenating agent, including orthopedics, plastic surgery, dermatology, and dentistry [20]. Sills et al. published the first study using intraovarian injection of calcium gluconate-activated autologous PRP [17]. They observed an increase in serum AMH and a decrease in FSH with at least one suitable blastocyst for cryopreservation in all four patients. Sfakianoudis et al. evaluated ovarian function after PRP in poor responders [19]. They reported increased number of mature oocytes and embryos reaching the cleavage stage in the post-PRP cycle. They reported a natural conception at 24 weeks, an uncomplicated healthy pregnancy at 17 weeks, and a successful live birth. In another study, Sfakianoudis et al. reported decreased cycle cancellation rates after PRP treatment [21]. Panda et al. performed a systematic review of PRP for patients with POR and POI [22]. They reported improved outcomes in terms of ovarian reserve parameters and IVF outcomes. More recently, Pacu et al. also reported increased AFC, and AMH, with decreased FSH and LH levels after PRP treatment [23].

Pregnancy rates in women with POR diagnosed according to the POSEIDON criteria (Groups 3 and 4) are reported to range between 12.7% to 35.5% [24]. In another analysis of 26,697 cycles, live birth rates in the first cycle in POSEIDON groups 3 and 4 were 14.73% and 6.58%, respectively [25]. The effect of PRP on pregnancy rates in POR patients has also been investigated. Melo et al. studied the effect of intraovarian PRP injection in 83 women with low ovarian reserve in a prospective controlled non-randomized study [26]. They found higher biochemical (26.1% vs. 5.4%, p=0.02) and clinical (23.9% vs. 5.4%, p=0.03) pregnancy rates in the PRP group. In another prospective non-randomized study (n=40), live birth rates following low dose ovarian stimulation and IVF in women treated with PRP was compared to controls [27]. There was a trend towards higher implantation and live birth rates in patients who underwent PRP treatment. Farimani et al. have investigated pregnancy rates in patients with POR diagnosed according to the POSEIDON criteria and have reported a 14.6% pregnancy rate among these patients [28]. In the current study, among 474 women in whom COH for IVF was attempted, 312 developed at least one cleavage embryo. While this could be considered a very encouraging result, only 83 women (30%) achieving a positive pregnancy test and only 54 women (19.5%) achieving SI/LB. This attrition could be due to a number of factors. The most likely cause is the age of the cohort, where 378 patients (74.1%) were 38 years or older, and 186 patients (36.4%) were older than 42 years. As expected, our findings do not suggest that PRP prevents the age related increase in aneuploidy.

The most abundant population of follicles in the ovary are the dormant primordial follicles, which consist of an oocyte surrounded by a single layer of granulosa cells [29]. Once activated, primordial follicles develop into primary follicles, secondary follicles, and ultimately may become antral follicles capable of producing a mature oocyte. Follicle activation might be induced through physiologic and non-physiologic pathways. Active substances such as growth factors and chemokines promote follicle activation and progression through stages of development. Some of these substances (transforming growth factor beta [TGF-β], insulin-like growth factor [IGF], platelet derived growth factor [PDGF], epidermal growth factor [EGF], basic fibroblast growth factor [bFGF], vascular endothelial growth factor [VEGF]), cytokines (interleukin 1 beta [IL-1β], IL-8) are present in PRP and may help explain follicular activation that occurs following intraovarian PRP injection [30, 31]. In addition, mediators released by platelets may reverse ovarian hypoperfusion and improve oxygen delivery and clearance of reactive oxygen species (ROS), resulting in improved recovery of follicles [32]. This effect has been suggested through recovery of mitochondrial function resulting in ploidy rescue in blastocysts. Supporting this hypothesis, Sills et al. have reported a healthy 46, XY pregnancy with IVF after intraovarian injection of PRP in a patient with 5 previous failed IVF due to 20 genetically abnormal embryos [33]. Within this context, our study provides some relevant clinical information. Our findings suggest that the improvements in AFC, FSH, and AMH levels after PRP might result from increased follicular recruitment and improved progression through follicular developmental stages. Also, the improvements in IVF parameters like number of oocytes, fertilization rates and the number of blastocysts per cycle may also be related to improvement in both oocyte quality and quantity.

In conclusion, intraovarian injection of autologous PRP might be considered in women with POR. The ideal population that may benefit from this approach can be summarized as patients <40 years old, with an FSH < 21.2 mIU/mL, AMH > 0.23 ng/ml, with at least one antral follicle, and a mean ovarian volume > 4.30 cm^3^. For wider clinical application, its clinical efficacy will need to be demonstrated in prospective randomized clinical trials. Until then, autologous PRP treatment should not be recommended as part of routine treatment in women with POR.

## MATERIALS AND METHODS

### Study design and patient selection

This was a prospective observational study of ovarian reserve parameters and IVF outcomes in women diagnosed with POR after undergoing intraovarian autologous PRP injection. The study was conducted at Acibadem Maslak Hospital, in Istanbul, Turkey, between January 1, 2020 and December 31, 2020. Women aged 30 to 45yo with a diagnosis of POR, a history of infertility for at least one year, and at least one ovary were included. Patients were diagnosed with POR using POSEIDON criteria. Patients with a history of malignancy, prior major lower abdominal surgery resulting in pelvic adhesions, anticoagulant use for which plasma infusion is contraindicated, and current or previous IgA deficiency were excluded. The study protocol was approved by the University’s institutional review board and ethics committee (ATADEK-2019-8/18) and registered at the http://www.Clinicaltrials.gov, (NCT04237909). All women included in the study signed a written consent form.

### PRP preparation

PRP was prepared by centrifugation as previously described, using a T-lab autologous platelet-rich plasma kit (T-Biotechnology Laboratory, Bursa, Turkey) according to the manufacturer’s instructions [34]. For each patient, a total of 20 ml of blood was collected under sterile conditions, and the tubes were centrifuged at 830 g for 8 minutes. Afterwards, a 16 G needle connected to a 5 ml syringe was inserted into the tube and advanced to the buffy coat layer. The PRP was drawn up with the syringe without removing the blood clot rich in growth factors. Approximately 2-4 cc PRP was collected from the first tube, and the second tube was processed in a similar way for a total of 4-8cc of PRP. The collected PRP solution was transferred to a separate tube and shaken gently for 30-60 seconds.

### Intraovarian injection

Intraovarian injection was performed in the operating room under conscious sedation within two hours of PRP preparation. PRP was injected using a 35 cm 17 G single lumen needle (Cook, USA), into at least one ovary transvaginally under ultrasound guidance. Although ovaries from advanced maternal age patients with POR may be small and fibrotic, PRP injection was achieved by creation of new planes within the ovary through distention and injection at multiple sites. After the procedure, the patients were taken to the recovery room and discharged home on the same day after observation for 30-40 minutes.

### Timing of PRP injection, patient assessment and follow-up

Intraovarian injection of PRP was performed within 10 days after completion of menstrual bleeding. On the same day, serum anti-mullerian hormone (AMH) and follicle stimulating hormone (FSH) levels and baseline antral follicle count (AFC) were determined prior to PRP injection.

After the PRP injection, all women were managed expectantly for 6 weeks to allow spontaneous pregnancy or menses. If menstruation did not start within 6 weeks, then a pregnancy test was performed and (if not pregnant) menstruation was induced hormonally using 2 mg estradiol valerate twice a day for 5 days and 2 mg estradiol valerate + 0.5 mg norgestrel twice a day for 5 days (Cycloprogynova, Bayer). If menstruation was delayed in the subsequent cycle(s), the same strategy was repeated.

At the beginning of the second menstrual cycle (day 2 to 4) after the PRP procedure, serum AMH and FSH levels and AFC were reassessed. Women who had at least one antral follicle more than they did at their initial evaluation began ovarian stimulation for IVF. Those whose AFC remained the same waited another cycle and were reevaluated. In the subsequent cycle, irrespective of the number of follicles compared to pre-PRP numbers, stimulation was started if at least one follicle was seen on each of the ovaries. In cases with a decrease in FSH or increase in AMH, but no antral follicles compared to basal measurements, stimulation was postponed to the next cycle.

### Controlled ovarian hyperstimulation and embryo transfer (ET)

Controlled ovarian hyperstimulation (COH) was initiated on the second or third day of the spontaneous or induced menstrual cycle. Gonadotropin stimulation was started at a dose of 300 IU recombinant FSH (Gonal F; Merck, or Fostimon; IBSA) and 300 IU human menopausal gonadotrophin (hMG) (Merional; IBSA). As soon as the dominant follicle reached a mean diameter of 14 mm, GnRH antagonist (Cetrotide; Merck) was initiated. When at least one leading follicle reached a mean diameter of 18 mm, 250 mcg recombinant chorigonadotrophin alfa (rHCG, Ovitrelle; Serono) was administered to induce final follicle maturation.

Oocyte retrieval was performed 34 h after rhCG administration. Four hours after the retrieval, oocyte denudation was done and all mature oocytes were inseminated via ICSI. Based on patient and physician preference, good quality embryos were either transferred on day 3 or day 5 after oocyte retrieval. For the patients who opted for preimplantation genetic testing for aneuploidy (PGT-A), all good quality blastocysts underwent trophectoderm biopsy followed by vitrification. PGT-A was performed by a commercial laboratory using an NGS-based assay (Veriseq PGS). Samples were reported as “euploid”, ”aneuploid”, ”mosaic”, or “no result”. Only euploid embryos were transferred after PGT-A.

Patients undergoing frozen embryo transfer (FET) began oral contraceptives on the second to fifth day of their cycle following stimulation, followed by subcutaneous injection of 3.75 mg leuprolide acetate depot (Lucrin; Abbott) in the midluteal phase. Oral estradiol (Estrofem; Novo Nordisk) was initiated for endometrial priming for five days at a dose of 4 mg daily and increased stepwise to 8 mg daily. After 14 days of estradiol, if the endometrial thickness was 8 mm or more and the progesterone level was below 1.0 ng/ml, then vaginal progesterone (Crinone gel 8% BID; Merck) was started twice a day with 17-hydroxyprogesterone caproate IM (Proluton Depot; Bayer) two times a week and FET was scheduled. If the endometrial lining was below 8 mm, an estradiol patch 7.8 mg (Climara; Bayer) was administered and patient was reassessed four days later. If the lining was still below 8 mm, then the cycle was cancelled. For fresh ET, daily vaginal progesterone (Crinone gel 8% BID; Merck) was used twice a day for luteal phase support starting on the day after the oocyte retrieval.

Pregnancy outcome was determined 12 days after ET by assessing serum ß-HCG level. Clinical pregnancy was defined by the presence of a gestational sac or fetal pole on transvaginal ultrasound after a positive pregnancy test. Sustained implantation was defined as the presence of an intrauterine pregnancy with cardiac activity at the time of discharge, around 12 weeks gestation.

### Statistical analysis

All data were analyzed using SPSS (SPSS-IBM 2.3, Inc., Chicago, IL, USA) and MedCalcsoftware version 18.11.6 (MedCalc Software, Broekstraat 52, 9030 Mariakerke, Belgium). Shapiro-Wilk test was used to assess the normality of data. For the matched samples of PRP before and after measurements, Paired Student’s t-test was used, while independent Student’s t-test was employed for independent groups. For continuous variables, the study results are summarized as mean ± standard deviation (SD). Categorical variables were presented as frequencies and percentages. p<0.05 was considered statistically significant. Receiver-operating curve (ROC) analysis with the area under the curve (AUC) was used to determine the levels of FSH, AMH, and AFC that predicted achievement of at least one embryo. As post-PRP outcomes have not yet been determined in cohort studies of adequate sample size, we could not perform a reliable power analysis before the initiation of the study.

## Supplementary Material

Supplementary Tables
