# Supplementary material for: Ovarian reserve parameters and IVF outcomes in 510 women with poor ovarian response (POR) treated with intraovarian injection of autologous platelet rich plasma (PRP)
Source: Aging (Albany NY). 2022 Mar 22;14(6):2513–23. doi: 10.18632/aging.203972 (PMC9004561; doi:10.18632/aging.203972)
Supplement: Supplementary Tables [file aging-14-203972-s001.pdf]

SUPPLEMENTARY TABLES

Supplementary Table 1. Characteristics of 22 women who had spontaneous pregnancy following PRP injection (mean ± SD) (range).

	mean ± SD (range)
Age	39.1 ± 4.4 (30-46)
Duration of infertility (years)	4.6 ± 3.3 (1-13)
Prior IVF attempts	1.7 ± 2.1 (0-8)
Number of menstrual cycles pregnancy achieved after PRP	2.0 ± 1.4 (1-7)
FSH (mIU/mL) prior to PRP	19.6 ± 23.9. (2.5-117.0)
AMH (ng/ml) prior to PRP	0.36 ± 0.27 (0.01-1.10)
AFC prior to PRP	2.8 ± 1.2 (1-4)

Supplementary Table 2. Clinical and IVF outcome parameters of women with POR who underwent intraovarian autologous PRP injection (mean ± SD).

	mean ± SD (range)
Patient Age (all women, n=510)	40.3 ± 4.0
Partner Age (all men, n=510)	41.4 ± 6.4
BMI (kg/m2)	25.7 ± 5.1
Duration of infertility (years)	7.4 ± 6.0
Number of previous IVF trials	2.8 ± 2.9
Days of stimulation (in women who underwent ovarian stimulation; n=474)	8.5 ± 2.7
Total gonadotropin dose (IU) (in women who underwent controlled ovarian hyperstimulation; n=474)	3820 ± 1213
E2 level (pg/ml) on the day of hCG (in women who underwent retrieval; n=427)	646 ± 542
